# Ascorbic Acid-Assisted Polyol Synthesis of Iron and Fe/GO, Fe/h-BN Composites for Pb^2+^ Removal from Wastewaters

**DOI:** 10.3390/nano10010037

**Published:** 2019-12-22

**Authors:** Denis Leybo, Marat Tagirov, Elizaveta Permyakova, Anton Konopatsky, Konstantin Firestein, Feruza Tuyakova, Dmitry Arkhipov, Denis Kuznetsov

**Affiliations:** 1 Department of Functional Nanosystems and High Temperature Materials, National University of Science and Technology “MISiS”, Moscow 119049, Russia; m1701562@edu.misis.ru (M.T.); feruzatuyakova@gmail.com (F.T.); arhipov.di@misis.ru (D.A.); dk@misis.ru (D.K.); 2 Laboratory of Inorganic Materials, National University of Science and Technology “MISiS”, Moscow 119049, Russia; permyakova.es@misis.ru (E.P.); konopatskiy@misis.ru (A.K.); 3 School of Chemistry, Physics and Mechanical Engineering, Science and Engineering Faculty, Queensland University of Technology (QUT), 2nd George St., Brisbane, QLD 4000, Australia; konstantin.faershteyn@qut.edu.au

**Keywords:** polyol synthesis, iron, Fe/GO composite, Fe/h-BN composite, ascorbic acid, lead ion removal

## Abstract

Iron powders and Fe/graphene oxide and Fe/boron nitride composites were synthesized by means of a polyol synthesis method. The effect of NaOH/Fe and ascorbic acid/Fe ratios on the characteristics of synthesized products were evaluated. The samples were characterized by X-ray diffraction, scanning and transmission electron microscopy, low-temperature nitrogen adsorption and Raman-spectroscopy. Ascorbic acid-assisted polyol synthesis resulted in the 10-fold decrease of the iron particles’ size and almost 2-fold increase of lead removal efficiency. The deposition of iron on the surface of graphene oxide lead to the formation of small 20–30 nm sized particles as well as bigger 200–300 nm sized particles, while the reduction in presence of boron nitride resulted in the 100–200 nm sized particles. The difference is attributed to the surface state of graphene oxide and boron nitride. Adsorption properties of the obtained materials were studied in the process of Pb^2+^ ion removal from wastewater.

## 1. Introduction

Polyol process is a unique method for the production of a number of different nanomaterials with narrow particles’ size distribution and well-defined morphology [1]. After the method was first introduced in the late 1980s, it attracted the attention of scientific groups due to its ability to produce finely divided metal powders with high crystallinity in a facile reproducible manner [2]. In the last decade, most research efforts have been directed towards the synthesis of noble metal-based systems by polyol method [3,4,5]. Such a high interest originates mostly from a high standard electrode potential of noble metals which can be reduced, even at room temperature.

In addition to noble metals, polyol synthesis of ferromagnetic materials was intensively studied due to the high crystallinity of the obtained particles, which results in higher saturation of magnetization [6]. Among the ferromagnetic materials, polyol synthesis of iron is the least studied process because of the difficulties of its reduction by polyol and low oxidation stability in ambient atmosphere. Nevertheless, recent studies showed that the reduction of iron is possible provided that synthesis conditions are followed precisely. It was reported by H. Chiriac et al. [7] that refluxing of iron sulphate solution in the presence of NaOH in ethylene glycol led to the formation of spherical particles with wide size distribution in a range from 60 to 400 nm.

One of the important aspects of iron powders’ synthesis by polyol method is the ability to control particles’ sizes in various ways. R.J. Joseyphus et al. [8] reported that the choice of polyol affects the size of iron particles. They found that the size decreases in the sequence of ethylene glycol > propylene glycol > trimethylene glycol. However, as a side effect of the decrease of particles’ size they observed substantial oxidation of iron particles during the process. Another approach to control the particles’ size during polyol process is the utilization of microwave radiation which enhances kinetics of particles’ formation. In the works of S. Komarneni et al. [9] and M.N. Nadagouda [10], authors used a microwave-assisted polyol synthesis approach to synthesize rod-shaped submicron iron particles. A successful approach to the synthesis of iron with particles’ sizes below 100 nm was proposed by R.J. Joseyphus et al. [11]. They used hexachloroplatinic acid as a nucleating agent for the synthesis of iron under heterogeneous nucleation conditions. Although the concentration of Pt ions was as low as 2 × 10^−7^ M, the scaling up of the technology will lead to appreciable cost effects. Thus, other routes to reduce particles’ sizes of polyol-synthesized iron should be considered. One such approach constitutes the addition of more powerful reducing agents to the polyol-iron salt system.

An ascorbic acid was widely used by different scientific groups for the synthesis of nanosized metal particles. Mostly, the attention was concentrated on the synthesis of noble metals and their alloys. Au [12,13], Ag [14,15], Pt [16,17], Pd [18] and different bimetallic [19,20] nanoparticles were successfully synthesized using ascorbic acid as the reducing agent. It was also shown that the ascorbic acid acts as a reducing agent in polyol synthesis of Rh [21], Ir [22] and Cu [23]. However, there is no information about the ascorbic acid-assisted polyol synthesis of iron in the literature.

Iron powders can be used in a variety of applications including magnetic materials, medicine, etc. [24]. One of the promising applications of iron in zero valent form is for wastewater treatment technology. It was shown previously that zero valent iron (ZVI) is efficient for the removal of recalcitrant organic compounds [25] and heavy metals [26,27]. We have shown that the ZVI can be used for the decomposition of azo dyes [28] and as a Fenton-like zinc removal catalyst [29]. In a majority of research works on this topic, borohydride-reduced iron was used as a waste treatment material. In the present study, for the first time, we employ polyol-synthesized iron for the removal of Pb^2+^ ions from water.

Deposition of metals on the surface of supports with extended surface area can lead to the increased efficiency of their functional utilization. It was shown in previous studies [30,31] that deposition of ZVI substantially enhances wastewater treatment due to the synergy between the activity of ZVI capable to reduce numerous ions in the solution and adsorption capacity of the support. However, there is scarce information in the literature about the use of Fe/GO and Fe/h-BN composites for water treatment and no information about the synthesis of such composites via polyol reduction.

The aim of the present work was to study the effect of ascorbic acid on the properties of the polyol-synthesized iron. In order to decrease the size of iron particles, we synthesized Fe/GO and Fe/h-BN composites by polyol reduction. We also studied the effect of sodium hydroxide concentration on the composition of produced powders and evaluated the efficiency of lead removal from aqueous solutions by the obtained materials.

## 2. Materials and Methods 

### 2.1. Materials

Ethylene glycol (EG, C_2_H_4_(OH)_2_, 99.8 wt.%), iron chloride tetrahydrate (FeCl_2_·4H_2_O, 99.8 wt.%), l-ascorbic acid (C_6_H_8_O_6_, 99 wt.%), and sodium hydroxide (NaOH, 98 wt.%) were purchased from RusHim, Moscow, Russia. Hexagonal boron nitride (h-BN) was purchased from Plazmotherm, Russia. Graphene oxide (GO) was purchased from Mineral, Russia, in the form of water suspension. Prior to the use, it was freeze-dried until the constant mass of the powder. 96 vol.% ethyl alcohol (C_2_H_5_OH) was used for washing procedures. MilliQ water and analytical grade lead nitrate salt (Pb(NO_3_)_2_) were used for wastewater treatment experiments.

### 2.2. Synthesis of ZVI Samples

ZVI samples were synthesized by modified polyol method as described elsewhere [11]. In detail, 1068 mg of FeCl_2_·4H_2_O were mixed with 0, 95 and 189 mg of C_6_H_8_O_6_ to obtain a C_6_H_8_O_6_/Fe molar ratio of 0, 0.1 and 0.2, respectively. The obtained mixture was dissolved in 54 mL of EG to prepare 0.1 M solution of iron. The solution was heated to 190 °C in an ambient air, and 4297 mg of NaOH, corresponding to an NaOH/Fe molar ratio equal to 20, was added within 2 min to the solution. Black precipitate was formed immediately after the sodium hydroxide addition. After cooling the mixture down to room temperature, the precipitate was separated from the liquid via centrifugation and washed with 350 mL of ethanol. The obtained powder was dried in vacuum at room temperature overnight.

In order to study the sodium hydroxide amount effect, samples without the addition of ascorbic acid were prepared in the same fashion as described above. The NaOH/Fe molar ratios of 10, 20, 30 and 40 were used for this series of samples. Composite Fe/GO and Fe/h-BN samples were prepared without the addition of ascorbic acid using an NaOH/Fe ratio of 20. For these experiments, a suspension of 200 mg of GO and h-BN in 54 mL of EG was prepared via ultrasonic treatment. The suspension was then used for the synthesis of ZVI particles as described above.

### 2.3. Characterization

Phase composition of samples was characterized by X-ray diffraction (XRD) on Difrey-401 equipment using Cr-K_α_ radiation (λ = 2.2909 Å) in the 2θ range from 20 to 120°. The diffractometer is equipped with position sensitive detector capable of simultaneous signal detection in the 2θ range of 58°. The crystallites’ size was estimated using the Scherrer equation, and the Si standard was used for instrumental broadening elimination. Specific surface area of samples was measured by low-temperature N_2_ adsorption on NOVA 1200e equipment at 77 K. Prior to the analysis, samples were degassed at 100 °C in vacuum for 3 h. The values of specific surface area were obtained using the Brunauer-Emmett-Teller (BET) method. Scanning electron microscopy (SEM) and transmission electron microscopy (TEM) were used to study the morphology of the synthesized samples. SEM images were obtained on JSM F7600 equipped with backscattered electrons and energy dispersive X-ray (EDX) detectors. Samples were embedded into water-based PELCO colloidal graphite. TEM was done on JEM-2100 instrument equipped with energy dispersive X-ray spectrometer. The samples for the analysis were ultrasonically dispersed in an absolute isopropyl alcohol medium and dried on a copper grid. Raman spectra were acquired using a Thermo Scientific DXR Raman microscope at an excitation wavelength of 532 nm. The Fe content in Fe/GO and Fe/h-BN samples was evaluated using an inductively coupled plasma mass spectrometry (ICP-MS) device. For the analysis, ca. 10 mg of sample was continuously stirred in aqua regia overnight at room temperature. The Fe concentration in the solution was used to calculate required Fe content. X-ray photoelectron spectroscopy (XPS) was done to analyze chemical composition on the surface of samples using Axis Supra spectrometer. A fitting process was conducted with CasaXPS software after the subtraction of Shirley-type background. C1s peak (284 eV) was used to calibrate XPS spectra.

### 2.4. Evaluation of Pb^2+^ Removal Efficiency

For the lead removal experiments, 25 mg of the iron-based material was added to 100 mL of 1 mM Pb(NO_3_)_2_ solution. The mixture was ultrasonicated for 10 min in a bath. Afterwards, the suspension was left in a shaker for 180 min at room temperature in an ambient atmosphere. The experiment was performed in natural pH of the solution. The 1 mL probes of the suspension were taken periodically, filtered through the 0.22 µm membrane filter and analyzed by means of inductively coupled plasma mass spectrometry on ICAP Q instrument. All the experiments were done in triplicates.

The samples were studied by means of ICP-MS using an X-Series II unit. The samples were diluted 1000 times and transferred to a 2% matrix HNO_3_ (Panreac AppliChem, Darmstadt, Germany) for matching calibration solution A (High Purity Standards, Charleston, SC, USA). To calibrate ICP, MS X-Series II unit Tune B solution was used. 2% solution of HNO_3_ was also used as control solution, and all element concentrations which were determined in blank solution were deducted from the results of the samples.

The removal efficiency of lead ions was calculated according to the equation:

$$\eta =\;\frac{C_{0}-C_{\tau }}{C_{0}}\cdot 100\%$$

where *C*_0_, *C_τ_*—initial concentration of lead ion and concentration at the time of experiment, respectively.

Lead removal capacity *q_e_* (mg·g^−1^) was calculated by the equation:

$$q_{e}=\frac{C_{0}-C_{\tau }}{m}\cdot V$$

where *m*—mass of the iron containing sample, *V*—volume of the solution used for the experiment.

## 3. Results

### 3.1. Phase Composition of the Samples

Phase composition is the main characteristic affecting functional properties of the materials. We have shown previously [32] that the synergy between zero valent iron and ferrihydrite phase can increase the efficiency of wastewater treatment. In the present work, we study the effect of sodium hydroxide and ascorbic acid on a phase composition of the resulting products obtained by polyol synthesis. It was reported [33] that hydroxyl group increases the rate of complexation between the metal ion and polyol. However, the deprotonation reaction of alcohols can result in sodium alkoxide formation [34]. Thus, it is crucial to find the NaOH/Fe ratio that allows to produce single-phase iron powder.

**Figure 1:**
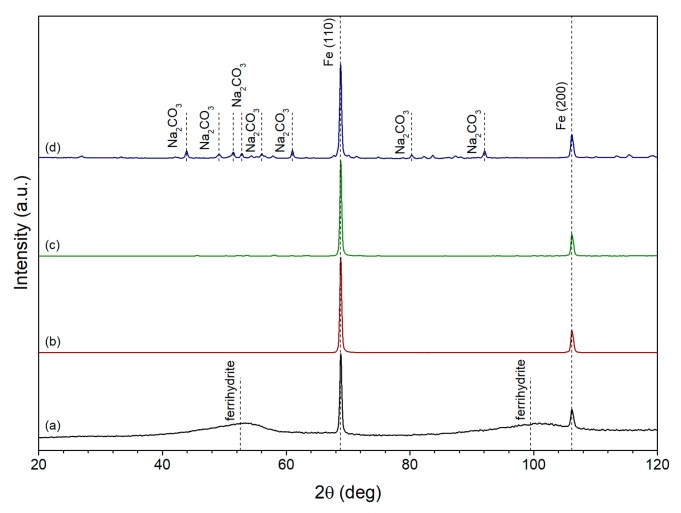
XRD patterns of iron samples produced via polyol synthesis using NaOH/Fe ratio equal to (**a**) 10; (**b**) 20; (**c**) 30; (**d**) 40.

In order to study the effect of the NaOH/Fe ratio on phase composition of polyol-synthesized iron, four samples with an NaOH/Fe ratio equal to 10, 20, 30 and 40 were prepared. XRD patterns of these samples are shown in Figure 1. The formation of body-centered cubic iron in the synthesized samples was confirmed by the presence of peaks at 68.8° and 106.2° (PDF card no. 06-0696). The two broad peaks, centered at 52.6° and 99.5°, appeared on the pattern of the sample obtained using the NaOH/Fe ratio equal to 10 (Figure 1a) and correspond to the formation of 2-line ferrihydrite phase [35]. The sample produced using NaOH/Fe ratio equal to 20 consisted of single iron phase (Figure 1b), while the increase of the ratio led to the formation of sodium carbonate impurities (Figure 1c,d). The obtained results showed that the optimum value of NaOH/Fe ratio was equal to 20, so this value was used in all subsequent experiments.

**Figure 2:**
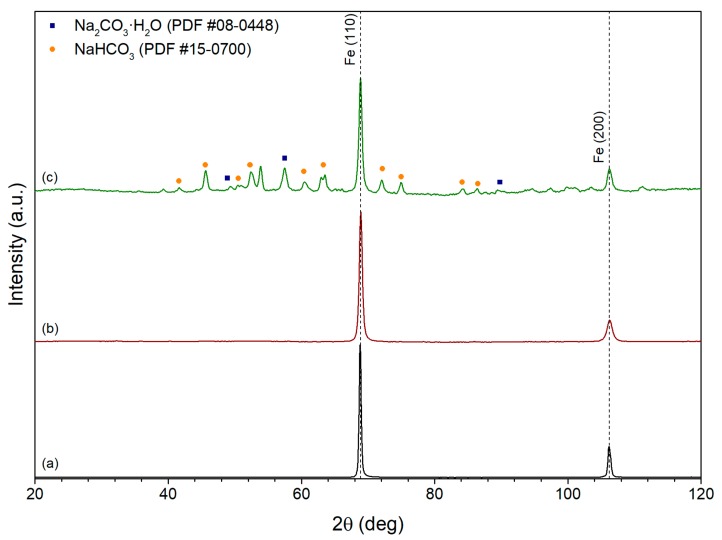
XRD patterns of iron samples produced via ascorbic acid-assisted polyol synthesis with C_6_H_8_O_6_/Fe ratio equal to (**a**) 0; (**b**) 0.1; (**c**) 0.2.

XRD patterns of samples produced by ascorbic acid-assisted polyol synthesis using C_6_H_8_O_6_/Fe ratios of 0, 0.1 and 0.2 are shown in Figure 2. As in the case of polyol synthesis, samples obtained with the addition of ascorbic acid consisted of iron phase. The addition of a small amount of ascorbic acid did not affect phase composition of the sample although the peaks corresponding to iron phase broadened (Figure 2b), which indicated the reduction of crystallites’ size. However, further increase of ascorbic acid content led to the formation of impurities (Figure 2c). When the C_6_H_8_O_6_/Fe ratio was bigger than 0.2, we did not observe any formation of iron-containing phases.

**Figure 3:**
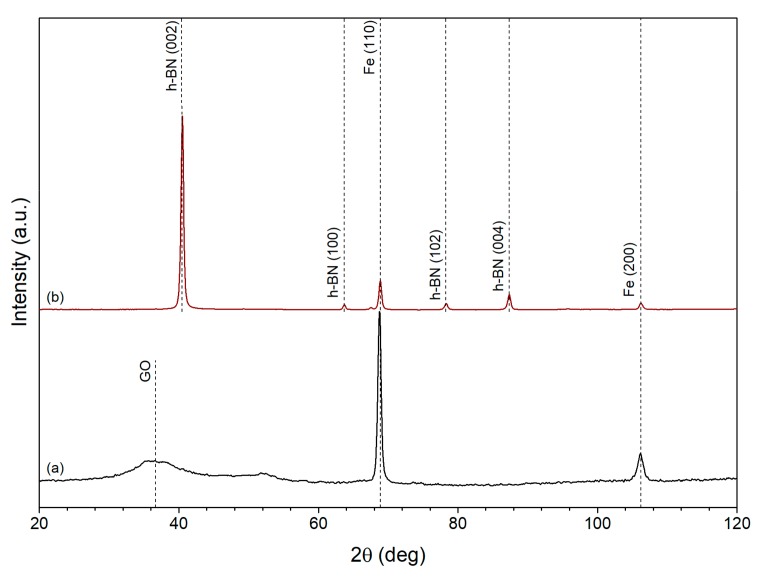
XRD patterns of (**a**) Fe/GO and (**b**) Fe/h-BN composite samples.

The reduction of iron ion by EG in the presence of GO and h-BN particles led to the formation of Fe/GO and Fe/h-BN composites. The corresponding XRD patterns are shown in Figure 3. It can be seen that no impurities formed during the synthesis. Only iron, GO and h-BN phases present in the samples, which is confirmed by the peaks at 68.8° and 106.2° (PDF card no. 06-0696) for iron, 36.7° for GO [36], and 40.5, 63.7, 78.3 and 87.4 for h-BN (COD card no. 2016171 [37]).

### 3.2. Morphology and Particles’ Size of the Samples

**Figure 4:**
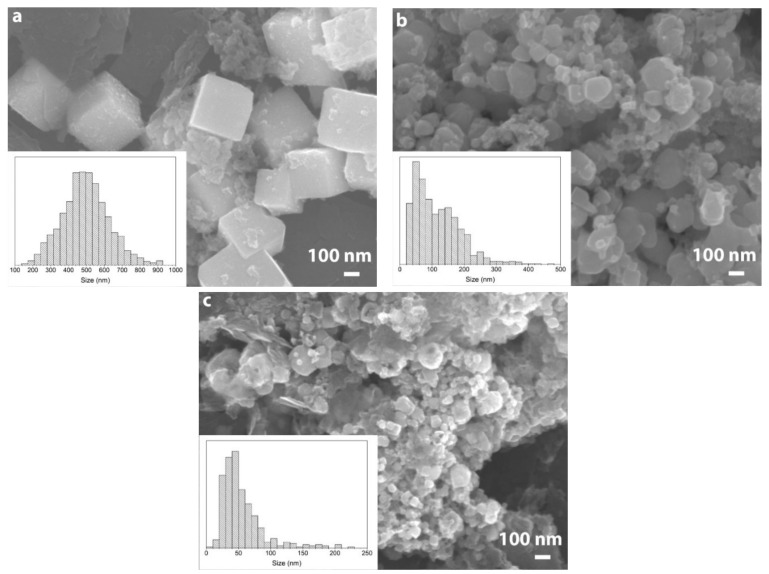
SEM images of polyol-synthesized iron samples produced with C_6_H_8_O_6_/Fe ratio equal to (**a**) 0; (**b**) 0.1; (**c**) 0.2.

**Figure 5:**
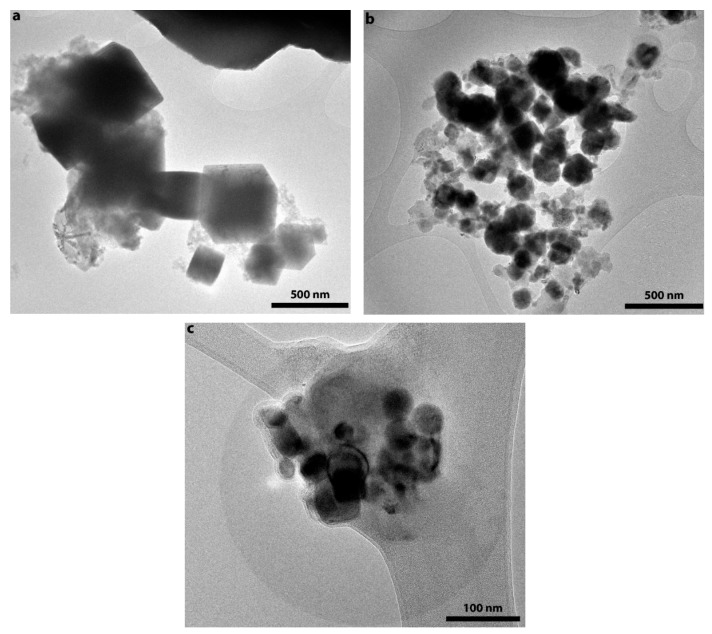
TEM images of polyol-synthesized iron samples produced with C_6_H_8_O_6_/Fe ratio equal to (**a**) 0; (**b**) 0.1; (**c**) 0.2.

In order to study the influence of ascorbic acid addition on the morphology of polyol-synthesized iron, SEM and TEM analyses of samples was done. The results are shown in Figure 4 and Figure 5.

One can see from the images that the addition of ascorbic acid significantly affected the morphology and size of the iron particles. Polyol synthesis of iron without addition of the ascorbic acid led to the formation of faceted cubic shaped particles with sizes in the range of 100–1000 nm (Figure 4a and Figure 5a). The addition of even small amounts of ascorbic acid resulted in smoothing of particles’ edges (Figure 4b and Figure 5b). Although there were cubic particles in the sample, their amount was vanishingly small. Additionally, particles’ size reduction down to 20–500 nm took place in this case. The particles’ size distribution was bimodal in nature with modes at ca. 50 and 150 nm. Further increase of ascorbic acid concentration exerted little influence on the morphology of iron particles. However, the size of the particles was decreased below 100 nm.

**Figure 6:**
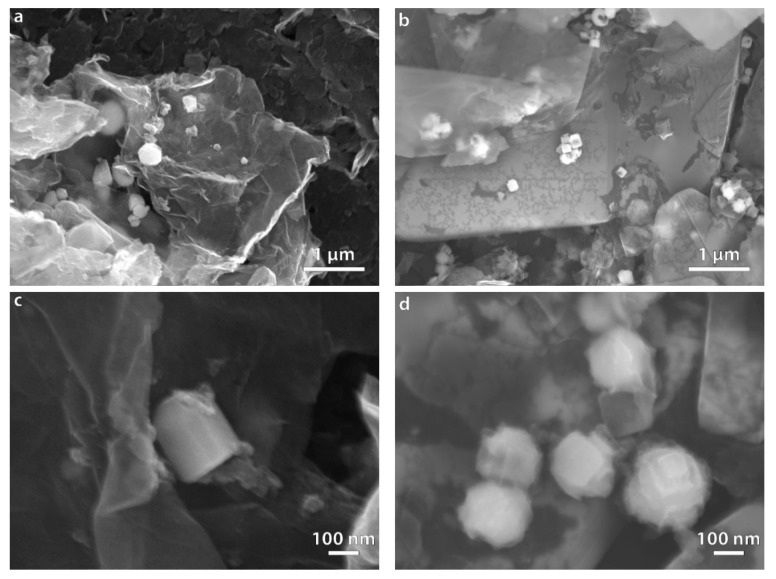
SEM images of composite polyol-synthesized samples (**a**) Fe/GO, ×20k; (**b**) Fe/h-BN, ×20k; (**c**) Fe/GO, ×100k; (**d**) Fe/h-BN, ×100k

**Figure 7:**
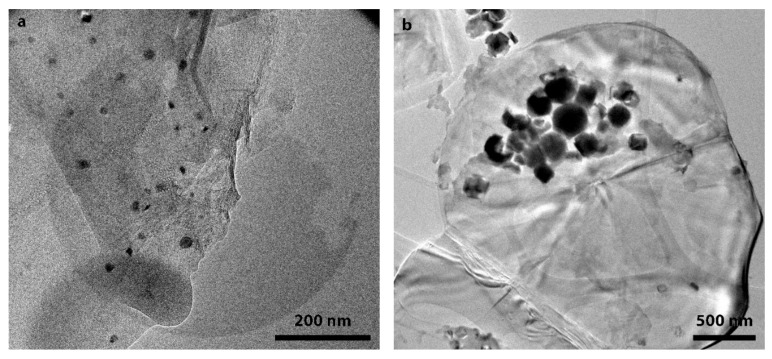
TEM images of composite polyol-synthesized samples (**a**) Fe/GO; (**b**) Fe/h-BN.

SEM and TEM images of composite Fe/GO and Fe/h-BN samples are shown in Figure 6 and Figure 7.

The polyol synthesis of iron in presence of GO led to the formation of particles with sizes in the range of 200–300 nm (Figure 6a). Most of the particles had irregular shape although some of them crystallized in the faceted form (Figure 6c). A characteristic feature of the synthesis was the formation of small 20–30 nm particles evenly distributed on the surface of GO sheets (Figure 7a). In case of Fe/h-BN composite synthesis, the morphology of particles differed markedly from the GO sample. Cubic and pyramid shaped particles with sizes of 100–200 nm were formed (Figure 6b,d). We have not observed the formation of smaller particles on the surface of h-BN (Figure 7b). Additionally, there was an obvious tendency of iron particles to form aggregates as evidenced by SEM and TEM results.

### 3.3. Raman Spectroscopy Analysis Results

**Figure 8:**
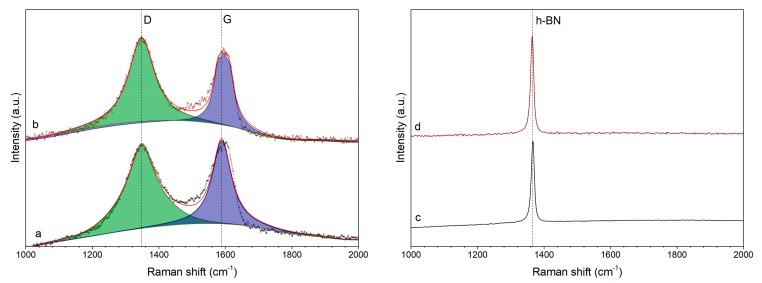
Raman spectra of (**a**) GO; (**b**) Fe/GO; (**c**) h-BN; and (**d**) Fe/h-BN samples.

In order to study the impact of polyol reduction on structural characteristics of iron-based composites, Raman spectra of GO and h-BN samples were measured in this work (Figure 8).

Raman spectra of GO containing samples had characteristic peaks at 1348 and 1589 cm^−1^ corresponding to D and G bands of graphene [38]. The I_D_/I_G_ ratios of these samples show the level of disorder in graphene and were equal to 1.42 for initial GO sample and 1.83 for Fe/GO sample. The obtained result indicates that polyol reduction of iron in the presence of GO flakes led to the increase of defects concentration in GO. In case of h-BN the only peak present on spectra of the two samples at 1365 cm^−1^ corresponded to boron nitride [39] and remained intact after the process of iron deposition.

### 3.4. Pb^2+^ Removal Results

**Figure 9:**
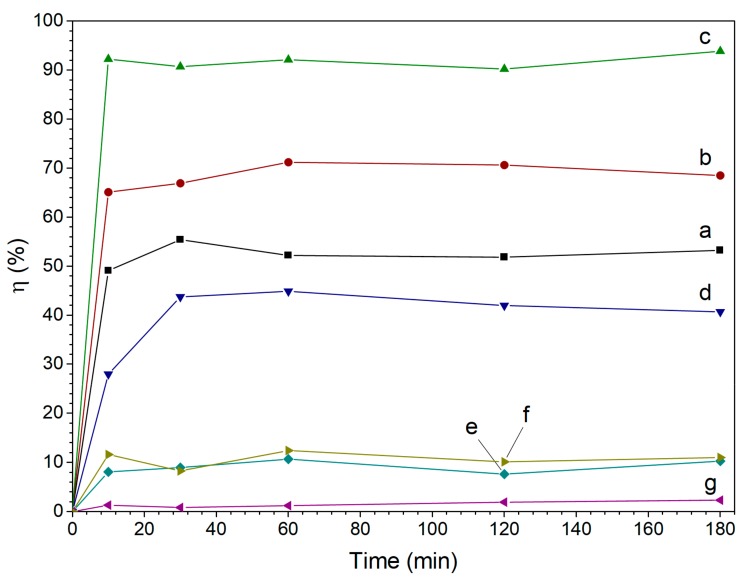
The efficiency of Pb^2+^ removal by polyol-synthesized iron using C_6_H_8_O_6_/Fe ratio equal to (**a**) 0; (**b**) 0.1; (**c**) 0.2; and by (**d**) Fe/GO, (**e**) Fe/h-BN composites, (**f**) pristine GO, (**g**) pristine h-BN.

The efficiency of lead ions removal as a function of time for samples produced using different ascorbic acid to iron ratio and Fe/GO, Fe/h-BN composite samples is shown in Figure 9. One can see from the plot that the removal of the most amount of lead took place in the first 10 min of the experiment. The removal capacity is increased in the order of Fe/h-BN < Fe/GO < Fe (C_6_H_8_O_6_/Fe = 0) < Fe (C_6_H_8_O_6_/Fe = 0.1) < Fe (C_6_H_8_O_6_/Fe = 0.2). Thus, the use of ascorbic acid during polyol synthesis of iron resulted in almost two-fold increase of lead ions removal efficiency.

### 3.5. XPS Analysis Results

**Figure 10:**
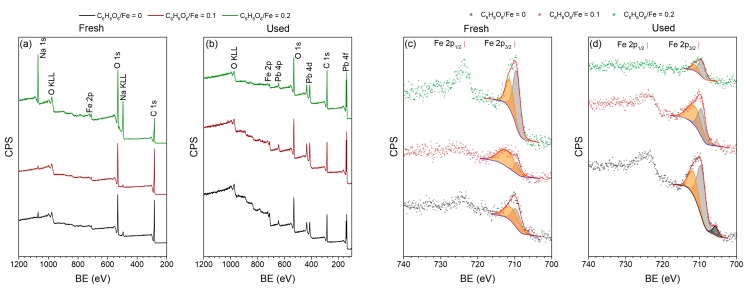

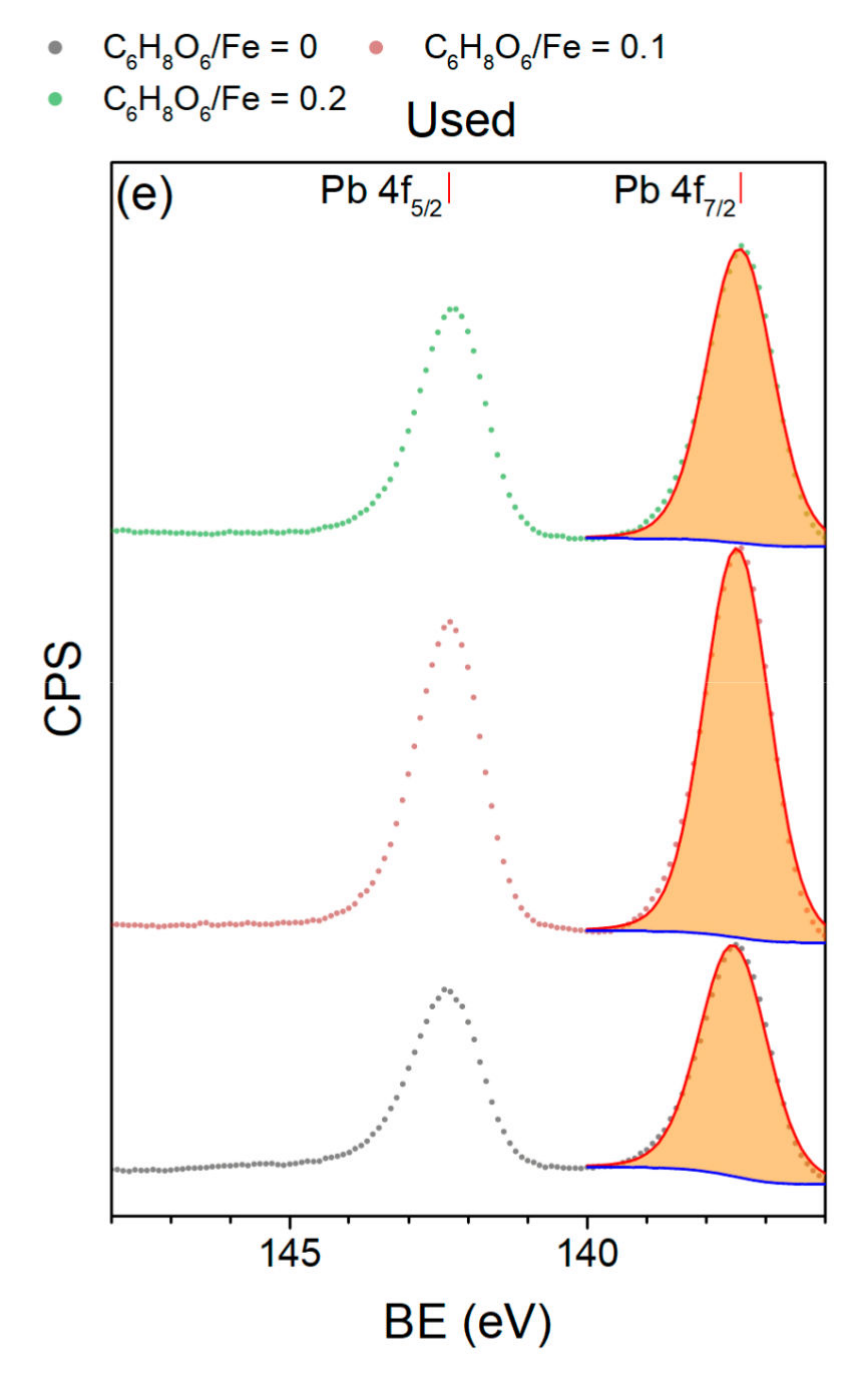
XPS spectra of fresh and used samples produced using different C_6_H_8_O_6_/Fe ratio. (**a**,**b**) Survey spectra; (**c**,**d**) Fe 2p high-resolution spectra; (**e**) Pb 4f high-resolution spectra

Surface chemical composition of samples produced using different ascorbic acid/Fe ratio was studied by means of XPS analysis. Survey spectra of samples are shown in Figure 10a. As can be seen from the figure, fresh-prepared samples consisted of Fe, O, C and Na elements which was confirmed by the presence of peaks at 710, 530, 284 and 1070 eV, respectively [30,40]. High resolution spectra of Fe 2p (Figure 10c,d) showed doublet at binding energies of 710 and 723.6 eV corresponding to Fe 2p_3/2_ and Fe 2p_1/2_ states, respectively [41]. The Fe 2p_3/2_ peak can be deconvoluted into two peaks centered at 709.5 and 711.3 eV which could be attributed to Fe^2+^ and Fe^3+^ [42].

XPS analysis of samples after the lead removal experiment revealed that sodium was absent on their surface while new peaks at 643, 412 and 137 eV, corresponding to Pb 4p, Pb 4d and Pb 4f, respectively, appeared on the spectra (Figure 10b). Pb 4f high-resolution spectra showed that lead was present in the 2+ oxidation state on the surface of samples which was confirmed by the Pb 4f_7/2_ peak at binding energy equal to 137.4 eV [43].

## 4. Discussion.

The characteristics of the samples calculated from the above analysis results are presented in Table 1.

### 4.1. Influence of NaOH/Fe Ratio on the Characteristics of the Samples

The addition of sodium hydroxide has significant influence on the polyol process. Elimination of this step from the iron production scheme results in the absence of precipitate formation. It was thought initially that sodium hydroxide addition led to the formation of iron hydroxide, Fe(OH)_2_, which was further decomposed to form iron oxide and metallic iron during disproportionation reaction [44]. This was proved to be incorrect during more recent works. R.J. Joseyphus et al. [8] showed that single phase iron could be synthesized using polyol process. The absence of iron oxide phases in the synthesized samples suggested that metallic iron was formed by the reduction of ions by polyol rather than the decomposition of iron hydroxide. The presence of hydroxyl ion can change the reduction potential of the redox couple present in the solution [45], thus making the process thermodynamically feasible. Another possible reason of NaOH action is the enhancement of acetaldehyde formation in the presence of OH^−^ ions, which is responsible for the reduction of metal ions during polyol synthesis [11]. Recently, Matsumoto T. et al. [46] found that the addition of sodium hydroxide to metal salt solution in EG results in the deprotonation of the latter with the formation of highly reactive monoanion EG species. The as-formed species were involved in the metal glycoxide synthesis, which is decomposed with the formation of metal particles. It was also noted [6] that the addition of NaOH may influence the size of the reduced particles as a result of the enhanced kinetics of the reduction.

**Figure 11:**
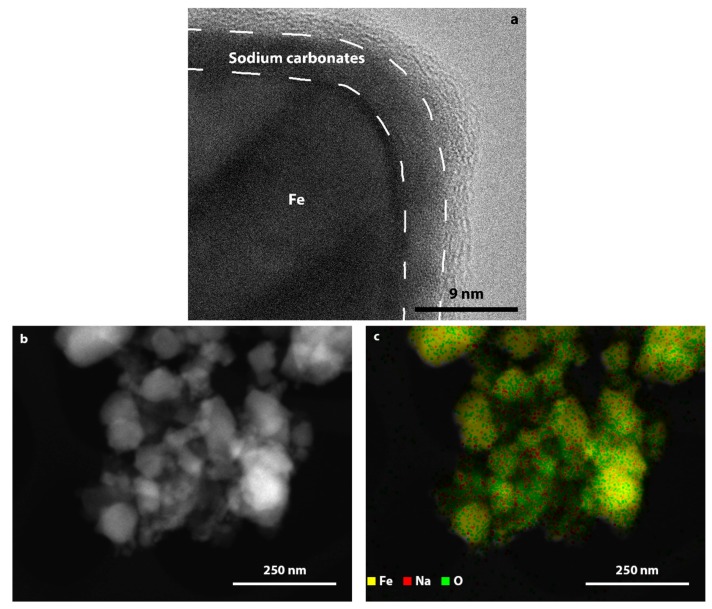
(**a**) HRTEM image of iron particle, (**b**) TEM image of iron particles and (**c**) corresponding element distribution map.

Our experimental results show that the crystallites’ sizes of iron calculated from XRD patterns did not change significantly with a change in the NaOH/Fe ratio (Table 1). This can be explained by the multiple step mechanism of metallic iron particles formation. The sodium hydroxide amount increase enhances the EG deprotonation kinetics. However, if the formation of iron-EG complex or the reduction of the complex is a rate limiting step, then the amount of sodium hydroxide will not influence the overall rate of iron ion reduction. Although the size of iron crystallites remained almost intact upon the increase of NaOH/Fe ratio, phase composition of samples differed significantly. The formation of a ferrihydrite phase is indicative of metallic iron corrosion in the presence of water [47]. The low crystallinity of the phase most probably means that the oxidation took place at low temperature during the washing step by the water and oxygen dissolved in ethanol. The increase of NaOH/Fe ratio resulted, at first, in the disappearance of ferrihydrite phase, while further addition of NaOH led to the formation of sodium containing impurities. Such relationship shows that products of the reaction between EG and sodium hydroxide deposited on the surface of iron particles and formed a passivation layer, protecting iron from spontaneous oxidation. High resolution TEM images of iron particles (Figure 11a) show that the surface of particles consists of a crystalline layer originated from sodium carbonates formed during a chemical reaction between NaOH and EG. This assumption is also confirmed by the results of XPS (Figure 10a) and EDX analysis (Figure 11b,c), which shows that sodium is evenly distributed on the particles of iron.

### 4.2. Influence of Ascorbic Acid on the Characteristics of the Samples

The size and shape of particles synthesized by polyol synthesis method depend on different parameters. The unique faceted morphology of particles originates from the selective adsorption of reacting species on the surface of seeds which changes the kinetics of particle growth in different directions [2]. Particles’ sizes of the obtained product depend on the rates of seeds formation and their growth. The rate of seeds formation is related to the kinetics of the reduction step, which, in turn depends on the stability of the initial salt used for the synthesis, reducing power of polyol and concentration of the reagents. Considering the abovementioned scheme, one can expect that the addition of more powerful reducing agents to the polyol process would result in the decrease of particles’ sizes due to the improvement in the reduction rate.

Our experimental results show that the addition of ascorbic acid to the EG solution led to an almost 10-fold decrease in median size of synthesized iron particles from ∼490 to ∼50 nm. Such dependence can be explained by the bigger reduction power of the ascorbic acid in comparison to the EG. Since the reduction of transition metals in the polyol process proceeds through the step of alkoxide complex formation, the relative reduction power of the compound can be estimated from its pK_a_ value. The pK_a_ value of EG equal to 15.1 at room temperature is greater than that of ascorbic acid (4.04) [48], which means that the ascorbic acid is more easily dissociated with the formation of active anions capable to form complexes with iron ion. Gradual decrease in particles’ sizes and the bimodal distribution of particles’ sizes in the sample with moderate ascorbic acid concentration indicates the simultaneous action of ascorbic acid and EG. Only at the ratio of C_6_H_8_O_6_/Fe equal to 0.2 does the reduction of iron ion occur mainly by ascorbic acid which is confirmed by monomodal character of particles’ size distribution curve for this sample. The formation of particles with shapes close to spherical with the addition of ascorbic acid can be explained by equal tendency of its adsorption on the iron seeds independent of crystallographic plane exposed on the surface.

### 4.3. Formation of Fe/GO and Fe/h-BN Composites

The formation of dispersed particles with decreased sizes on the surface of the supports is mainly attributed to the heterogeneous nucleation which is energetically favorable to homogeneous formation of seeds in the presence of solid support particles. The main factors affecting the deposition process are the state of the support particles’ surface, the specific surface area of the support and the parameters of synthesis procedure specific to the synthesis method. It was shown previously [49] that the increase of oxygenated groups concentration on the surface of GO result in the decrease of Pt particles’ sizes in a Pt/rGO system. Polyol synthesis of composite samples is also affected by the pH and temperature of the reaction. Chang-Chen Chou et al. [50] have shown that the dependence of Pt particles’ size deposited on GO on pH value possesses extreme character. It was shown that the increase of reaction temperature and decrease of pH tend to give smaller particles deposited on GO.

Although we did not perform a systematic study of different parameters effect on the properties of Fe/GO and Fe/h-BN samples, our preliminary results are valuable since there was no research works found regarding the polyol synthesis of these systems in the literature. Our experimental results show that the reduction of iron by EG proceeds differently in the presence of GO and h-BN particles. The formation of 20–30 nm sized iron particles on the surface of GO indicates the strong interaction between GO surface and intermediate species formed during the reduction process. Thus, prior to the reduction step, the chemisorption of an iron-EG complex on the surface of the GO particle occurs. However, the formation of 200–300 nm particles points to the low concentration of active centers on the surface of GO responsible for Fe-GO interaction. After the formation of small particles, further reduction takes place on the surface of as-formed iron. The absence of small particles on the surface of h-BN can be explained by much weaker interaction of h-BN surface with iron intermediates. The difference between these two cases can be explained by the surface state of GO and h-BN. It is well known that the GO sample contains hydroxyl and carboxyl groups on its surface [51]. These groups have higher energy in comparison with their surroundings and play the role of adsorption sites for the solute molecules. The adsorbed molecules are reduced by EG during the process leaving defects in the structure of GO characteristic to the reduced graphene oxide as evidenced by the increased I_G_/I_D_ ratio [52]. The h-BN surface is almost free of high energy bonds and there is weaker chemical interaction between reduction reaction participants and the surface of boron nitride. Moreover, iron particles are mostly found in aggregates which indicates preferable Fe-Fe interaction in this system. The unchanged Raman spectrum of h-BN sample before and after polyol synthesis of iron also suggests minimum involvement of boron nitride in the process. Nevertheless, we believe that additional surface treatment of h-BN can lead to the increased interaction with iron during polyol reduction. Thus, we have shown previously [53] that controllable oxidation of h-BN surface with oxygen results in the formation of Ag/h-BN nanohybrids. However, further studies in this direction have to be done with an iron reduction process to confirm this assumption.

### 4.4. Lead Ions Removal Efficiency

The lead ions removal trend found in the present study is well correlated to the physicochemical characteristics of Fe samples used in this work. Among the samples produced with different ascorbic acid/Fe ratio, the best performance possesses the sample with the least particles’ sizes and the biggest specific surface area. The reduction of the efficiency of Fe/GO and Fe/h-BN samples can be explained by the reduced content of iron per mass unit of the sample. Higher efficiency of Fe/GO sample is attributed to its higher surface area and Fe content as well as to the presence of small iron particles on the surface. One can also notice that the Pb^2+^ removal capacity calculated on the basis of iron content in these samples is substantially higher in comparison to almost all iron samples synthesized without the use of the support (Table 1). The removal efficiency of Fe based composites is also substantially higher than that of pristine GO and h-BN materials (Figure 9) which demonstrates that the efficiency of iron use can be increased by its deposition on the support.

The removal of lead ions from aqueous solutions by ZVI particles can proceed through various mechanisms, namely, adsorption, reduction and precipitation [43]. The reduction potential of Fe^2+^ (−0.44V) is lower than that of Pb^2+^ (−0.13V) which means that iron metal can reduce lead ions from the solution with the formation of insoluble Pb^0^ species [54]. Our experimental results show that lead is present in the form of Pb^2+^ state on the surface of used ZVI samples (Figure 10e) which suggests that the reduction is not the main mechanism of lead ions removal in our case. XPS results of ZVI samples before and after wastewater treatment experiments showed that the surface of fresh samples was covered by Na-containing phases which dissolved after the lead removal experiment. According to XRD analysis results, sodium was present in the form of carbonates, which upon exposure to aqueous media could be hydrolyzed inducing local increase of pH in the proximity of ZVI surface. This process was accompanied by the precipitation of lead hydroxide on the surface of ZVI.

## 5. Conclusions

Iron powders and Fe/GO and Fe/h-BN composites were successfully synthesized by means of polyol reduction in ethylene glycol at ambient atmosphere. The NaOH/Fe ratio did not affect the sizes of iron crystallites substantially, but did result in the formation of a passivation layer mainly composed of sodium carbonates. These carbonate species formed during the reaction between sodium hydroxide, and ethylene glycol protected the iron particles from irreversible oxidation during further technological operations and storage. The addition of ascorbic acid to the synthesis medium led to the 10-fold decrease in the size of iron particles from 492 to 46 nm. The diminution of particles’ size is attributed to the enhanced kinetics of iron formation due to the higher reducing power of ascorbic acid in comparison to ethylene glycol. During the polyol reduction, cubic-shaped particles formed while the addition of ascorbic acid led to the formation of particles with shapes close to spherical. The reduction of iron in the presence of GO and h-BN particles in the EG proceeded differently. Small 20–30 nm, along with bigger 200–300 nm particles, formed in case of GO while the reduction of iron in the presence of h-BN lead to the formation of 100–200 nm particles. This dissimilarity is attributed to the different surface state of the supports which resulted in weaker interaction of reduction reaction intermediates with h-BN. Also, we observed the reduction of GO particles during the process while the h-BN structure remained unchanged.

Lead removal efficiency increased almost two-fold as a result of the ascorbic acid assisted polyol synthesis of iron. Deposited on the surface of GO and h-BN iron samples showed inferior efficiency; however, the optimization of the deposition process can lead to the increase of the composites’ performance.

## Figures and Tables

**Table 1:** Characteristics of Fe and Fe/GO, Fe/h-BN samples.

NaOH/Fe	C_6_H_8_O_6_/Fe	Support	Fe Content, mg/g	Surface Area, m^2^/g	Fe Crystallites’ Size, nm	Median Particles’ Size, nm	Pb^2+^ Removal Capacity, mg/g
10	0	-	-	-	326	-	-
20	0	-	-	5.6	352	492	441
30	0	-	-	-	387	-	-
40	0	-	-	-	369	-	-
20	0.1	-	-	9.4	86	102	568
20	0.2	-	-	20.3	39	46	778
20	0	GO	168.9	43.6	37	-	337 (1995 *)
20	0	h-BN	128.7	13	159	-	85 (661 *)

* the value calculated per gram of iron in the sample.
